# The HTLV-1 latency-maintenance factor p30II activates p53-dependent metabolic effectors to promote oncogene-activation and aberrant lymphoproliferation

**DOI:** 10.1186/1742-4690-12-S1-P10

**Published:** 2015-08-28

**Authors:** Robert Harrod, Megan Romeo, Aditi Malu, Tetiana Hutchison, Averi White, Rachel Gardner, Olivia Nguyen, Catherine Hazen, Neha Rao

**Affiliations:** 1 Laboratory of Molecular Virology, Department of Biological Sciences and The Dedman College Center for Drug Discovery, Design & Delivery, Southern Methodist University, Dallas, Texas, USA

## 

The human T-cell leukemia retrovirus type-1 (HTLV-1) transforms CD4+ T-lymphocytes and causes adult T-cell leukemia/lymphoma (ATLL), an aggressive hematological malignancy that is refractive to most anticancer treatments. The highly-conserved pX sequence of HTLV-1 encodes three latency-maintenance factors: p30II, p13II and Hbz, which suppress proviral gene expression and help HTLV-1-infected cells evade detection by host immune responses as a prelude to neoplastic disease. We have previously demonstrated that p30II enhances the transcriptional and oncogenic functions of the c-Myc oncoprotein and induces aberrant lymphoproliferation through molecular interactions with the TIP60 acetyltransferase. Our recent studies have demonstrated the cooperation between p30II and c-Myc is dependent upon the induction of p53-dependent pro-survival signals. The p30II protein activates p53 and induces expression of p53-dependent metabolic effectors, including the Tp53-induced glycolysis and apoptosis regulator (TIGAR). Acute and lymphoma-stage ATLL clinical isolates frequently over express c-Myc and contain wildtype p53. We therefore hypothesize the induction of p53-dependent pro-survival signals by p30II could promote c-myc oncogene-activation during retroviral carcinogenesis by preventing c-Myc-induced cytotoxicity and apoptosis. Indeed, we have found that lentiviral-p30II induces mitochondrial expression of TIGAR and prevents the intracellular accumulation of c-Myc-induced reactive oxygen species (ROS) and inhibits oncogene-induced cellular senescence. Knockdown of TIGAR expression, using a small-interfering RNA (siRNA-tigar), inhibited the ability of p30II to suppress c-Myc-induced cytotoxicity. A panel of primary ATLL patient tumor lymphocytes exhibited over expression of TIGAR which correlated with c-Myc deregulation, compared to donor hu-PBMCs. Furthermore, we have shown that siRNA-knockdown of TIGAR expression sensitizes HTLV-1-transformed SLB1 lymphoma cells to oncogene-induced ROS and causes cellular senescence, suggesting siRNA-inhibition of TIGAR may be a plausible approach to sensitize ATLL tumor lymphocytes to anticancer chemotherapy drugs that induce oxidative damage. Our studies reveal a paradoxical role for p53-dependent metabolic effectors in promoting oncogene-activation by HTLV-1 p30II, which could promote proviral replication and leukemic disease progression.

